# Satellite-based monitoring of groundwater depletion in California’s Central Valley

**DOI:** 10.1038/s41598-019-52371-7

**Published:** 2019-11-05

**Authors:** Donald W. Vasco, Tom G. Farr, Pierre Jeanne, Christine Doughty, Peter Nico

**Affiliations:** 1 Energy Geosciences Division, Lawrence Berkeley National Laboratory, University of California, Berkeley, California 94720 USA; 2 Jet Propulsion Laboratory, California Institute of Technology, Pasadena, California USA

**Keywords:** Hydrogeology, Geophysics

## Abstract

Range change data, obtained from Synthetic Aperture Radar satellites, form the basis for estimates of aquifer volume change in California’s Central Valley. The estimation algorithm incorporates a function penalizing changes far from known well locations, linking the aquifer volume changes to agricultural, industrial, and municipal pumping within the Tulare basin. We show that the range changes are compatible with the hypothesis that the source of aquifer volume changes are variations in effective pressure around documented wells. Specifically, inclusion of the well distance penalty does not degrade the fit to the observations, inversions with and without it both give variance reductions of 99.6%. The patterns of aquifer volume change vary significantly from the drought year, between October 2015 and October 2016, to a wet year in 2017, and into 2018, a year with near average rainfall. The 2.3 million acre-feet of estimated volume reduction, a lower bound on the amount of water extracted from the basin between October 2015 and 2016, agrees with independent estimates of 1.8 and 2.3 million acre-feet. The aquifer volume reduction is also compatible with a loss of 3.1 km^3^ (2.5 million acre-feet) in groundwater volume derived from Gravity Recovery and Climate Experiment (GRACE) satellite data.

## Introduction

Estimating groundwater usage and the loss of aquifer storage capacity is a global challenge, hampered by a lack of systematic and quantitative monitoring. Even in highly developed agricultural areas, such as the Central Valley of California, active water wells are not currently required to monitor or report extracted volumes of water to any public agency^1^. The absence of such information makes it difficult to develop sustainable management policies for over-subscribed groundwater basins. Monitoring is critical in assessing the loss of storage capacity for a given aquifer and in determining the rate of groundwater depletion, at a scale corresponding to the needs of the agencies responsible for groundwater management, from a few kilometers to tens of kilometers. Furthermore groundwater withdrawal can produce significant surface deformation, leading to extensive damage of infrastructure, such as bridges, roads, pipelines, and canals. Given the need for the safe and sustainable management of groundwater resources in California, it is desirable to have cost-effective and timely bounds on aquifer volume changes at an appropriate spatial scale.

In light of the lack of detailed and wide-spread quantitative measurements of water withdrawal, as provided by comprehensive and accurate flow meter data, several efforts have been made to estimate groundwater usage. Unfortunately, each of these approaches suffers from serious short-comings that limit their usefulness. For example, observations of water levels in a distributed network of monitoring boreholes can indicate the status of critical aquifers^2^. However, due to economic considerations such boreholes are usually shallow, sparsely distributed, and only record changes in water level. Therefore, they may not accurately reflect the health of deeper aquifers nor provide information on localized areas of high water use. Electrical power demand has served as a proxy for pumping activity^3^, but such measures are indirect and may be influenced by the depth of the aquifer and the efficiency of the pumps, among other variables. Furthermore, such data are often aggregated over entire townships and intermittent, lacking adequate resolution for systematic monitoring. Crop type and crop water-intensity, along with well density, have been used to estimate the expected water usage, and these factors correlate with observed surface subsidence^4,5^, and observed changes in groundwater storage^6^. Such an approach can be combined with high-resolution Landsat satellite images to determine the distribution and health of crops more accurately^7^. The calculations depend upon several classes of observed and estimated parameters, such as surface water deliveries, evapotranspiration, and rainfall, among other properties, leading to sizable uncertainties. Satellite-based observations of time-variable gravity, such as those provided by the Gravity Recovery and Climate Experiment (GRACE) mission^8^, are sensitive to variations of total water storage^1^ and have been used to estimate basin-scale changes in water mass over time^9^. Unfortunately, the spatial resolution is not sufficient to monitor localized demand at the scale of a collection of water users. The result is a wide gap in the scales of monitoring, between the point observations of the wells and the basin-wide scale of GRACE data.

Geodetic techniques, measurments of ground deformation, provide a suite of observations that may be used to characterize water volume changes at intermediate scales between GRACE and well data. It has long been recognized that groundwater withdrawal may lead to observable surface deformation, due to the changes in effective pressure within an aquifer^10^. More recently, satellite-based Interferometric Synthetic Aperture Radar (InSAR) data have been used to image surface deformation associated with groundwater withdrawal and replenishment^11–13^. Such techniques have proven useful in California’s Central Valley and identify large-scale subsidence due to groundwater pumping and potential hazards to existing infrastructure^14–17^. Because the satellites are imaging displacements that occur at the Earth’s surface, techniques such as InSAR provide improved spatial resolution in comparison to satellite-based gravity observations. However, ground deformation itself is an indirect measure of the changes within an aquifer and additional analysis is required in order to extract quantities needed by water managers. Specifically, one must solve an inverse problem in order to relate the InSAR observations to the aquifer volume changes^18–20^. Additional parameters describing the poroelastic or poroplastic response of the medium are required to relate aquifer structural volume changes to changes in the volume or the mass of water in the aquifer^21^. This last step typically requires data that are difficult to gather and are likely to be unavailable, including the minimum effective pressure that the aquifer has ever been subjected to^21–23^, though there have been efforts to reformulate the problem to make inferences directly from InSAR range change, water level, and geological data^24^.

**Figure 1:**
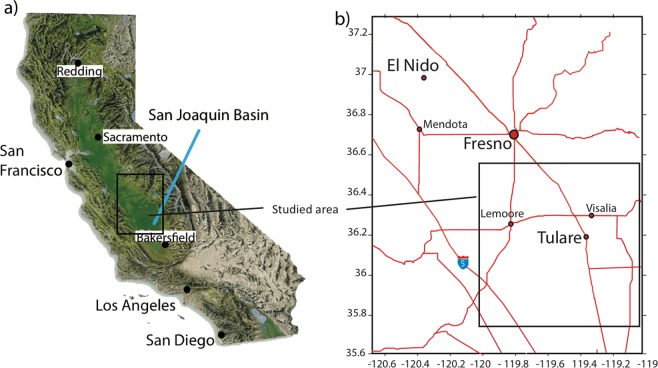
(**a**) Location of the study area within California. (**b**) Detailed position of the study area with respect to towns within the Central Valley of California.

Here we discuss the inversion of InSAR observations to infer aquifer volume change, the net impact of all withdrawal and recharge processes, throughout the Tulare basin in the southern San Joaquin Valley of California (Fig. 1). This work should be viewed as the initial step in an effort to develop bounds on the changes in groundwater volumes over time. These volume changes are assumed to be driven primarily by pumping from the aquifers, as well as by effects such as local and regional recharge, and long term compaction in clay formations. In order to tie the aquifer volume changes to agricultural, industrial, and municipal uses, we incorporate the publicly available digital map of state wells provided by the California State Water Resources Control Board, as a constraints in our inversion algorithm. This database, made available in early 2017, is an outgrowth of changes motivated by the Sustainable Groundwater Management Act (SGMA), and a mandate requiring publicly accessible reports for any well constructed in California within 60 days of its completion,

The estimated volume decreases presented below, while related to the changes in stored groundwater, are at best lower bounds on the volume of water removed from aquifers in the Tulare Basin. There are several complicating effects that make it difficult to take the next step, which is to estimate actual water volume changes at depth. First, in shallow unconfined aquifers, changes in the water table may not lead to detectible surface deformation, particularly in the northeastern corner of our study area where the sediments are thought to be fairly coarse^5^. In that area, changes in effective pressure may not lead to observable surface displacement and fluid withdrawal will be under-estimated. In confined aquifers, coarse sediments can also support some portion of the load transferred during the fluid pressure reduction due to groundwater pumping. Even for fine grained sediments, the inelastic volume reduction will not be equal to the fluid volume reduction due to the expansion of the fluid and the visco-elastic and plastic behavior of the porous matrix. Regardless, the spatial distribution of volume change can be used to identify regions and aquifers in the Tulare Basin that might be experiencing notable losses or gains in groundwater volume due to overuse or improved management, respectively. This could provide a tool to allow responsible agencies to assess one of the key undesirable results outlined in California’s groundwater management plan, namely’significant and unreasonable reduction of groundwater storage’. In addition, the spatial variation in volume changes can be used, along with a geomechanical simulator, to calculate full three-dimensional displacements within the overburden in order to estimate potential hazards to existing infrastructure, such as adjacent wells, gas pipelines, and canals. Localized horizontal displacements, which may not be detectible using InSAR, can pose significant problems in such structures.

## Results

Here we describe the outcome of our analysis of the InSAR data using the inversion techniques described in the Methods section. As noted above, we have structured our methodology to favor models that contain volume changes near known well locations. We find that it is possible to construct a model that both satisfies the InSAR observations to within their expected errors and honors the distribution of wells, both laterally and in depth. To begin this section, we motivate our effort with an illustration of a conventional approach to the inversion of the range change data that does not account for the distribution of wells, showing that it leads to a model containing volume changes that are far from locations known to have experienced fluid withdrawal.

The focus of our study is the aquifer volume change within the Tulare Basin, one of the most important agricultural areas in the world^16^. The Tulare Basin makes up the southern portion of California’s Central Valley, a 700 km long, 80 km wide sedimentary basin bounded to the west by the Coast Range and to the east by the Sierra Nevada Mountains (Fig. 1). The Tulare Basin’s areal extent is approximately 14,000 km^2^. The maximum sediment depth along the axis of the Central Valley is 16 km, but the bottom of fresh-water bearing formations in the Tulare Basin, the focus of this study, is at 750 m depth and dips to the southwest (see Supplementary Fig. S1). These deposits consist of sand and gravel interbedded and mixed with clay and silt and are quite heterogeneous, reflecting highly variable depositional environments^4^, and resulting in few regionally mapable deposits. One exception is the distinctive, laterally extensive low-permeability Corcoran Clay, a 10 to 30 m thick Pleistocene lake-bed deposit. This regional feature lies at depths ranging from 90 to 260 m in the Tulare Basin, and separates higher-permeability, semi-confined aquifers above it from lower-permeability confined aquifers below. Porosity and permeability values range from 37–48% and 1–10 Darcies for aquifers, and from 36–51% and 1 milli-Darcy for aquitards^4^.

**Figure 2:**
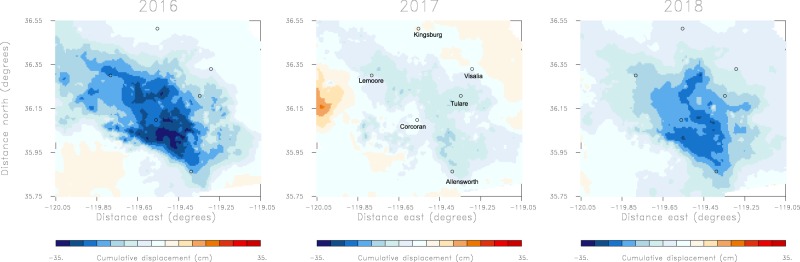
Line-of-sight displacements from Sentinel InSAR observations. (Left panel) Displacement along the line-of-sight to the Sentinel satellite that occurred between October 2015 and October 2016 [Water year 2016]. A positive displacement is associated with surface uplift while negative displacement signifies subsidence. The displacement is cumulative for a given water year, obtained by adding incremental changes from successive satellite passes. (Central panel) Line-of-sight cumulative displacement observed between October 2016 and October 2017. (Right panel) Cumulative line-of-sight displacement measured between October 2017 and October 2018. Several Central Valley towns are indicated by the open circles in the figure and are labeled in the middle panel.

Groundwater usage has led to extensive surface deformation throughout the southern portion of the California Central Valley that is observed by synthetic aperture radar (SAR) monitoring from orbiting satellites^14–16,25,26^. SAR monitoring, with a spatial resolution of tens of meters, can determine localized changes, in contrast to GRACE gravity observations that have associated spatial averaging kernels of a few hundred kilometers^8,27^. The observed deformation is evident in the three one-year intervals of range changes, changes in the distance to the satellite over time, for 2016, 2017, and 2018, shown in Fig. 2. The panels display range changes that occur between October of the preceding year to October of the given year. Such a time interval captures the entire rainy season and does not split the winter rains between years. Note that this is the definition of a water year according to the U. S. Geological Survey. There are significant variations in the magnitudes of the displacements for each year, reflecting the effects of the continuing drought in 2016, followed by significant rainfall in 2017, and a year with moderate precipitation in 2018. Using these data we can infer the spatial distribution, both laterally and in depth, of volume changes within the aquifers comprising the Central Valley hydrological system. As shown in the Methods section, assuming that the overburden, including those portions of the aquifer not undergoing fluid depletion, behave elastically over the time interval between satellite passes, we can set up a linear relationship between aquifer volume change and observed InSAR range change. Note that we are not assuming that the actively deforming volume of the aquifer behaves elastically. Such volume changes can be due to inelastic deformation, particularly in deformable clay layers, as well as due elastic deformation that is often observed in sand bodies or in formations where the water pressure is not lowered below historical levels^22,28,29^.

The forward problem consists of calculating surface displacements from specified volume changes, while the inverse problem is concerned with estimating the aquifer volume changes from observed surface displacements. One difficulty associated with inverse problems is the loss of resolution with depth within the Earth. That is, a volume change with a horizontal extent of tens of meters, at a depth of hundreds of meters, will spread to a surface anomaly that is hundreds of meters wide. A distribution of such anomalies at depth will like-wise spread and merge into a large-scale surface feature. This smoothing effect leads to an ill-posed inverse problem when trying to infer volume changes at depth from a set of surface observations. A related issue is the non-uniqueness, whereby multiple models of aquifer volume changes lead to the same set of surface displacements. The problem is particularly acute when volume changes can occur at various depths, as there can be trade-offs between the changes at different depths within the aquifer^20^.

The conventional approach to stabilize the inverse problem is to add penalty terms that seek to minimize the roughness of the model and the size of the allowed volume changes^30^, as in the function *P*(v) given by equation (4) in the Methods section. Such terms have the undesirable effects of concentrating the volume changes at, or near, the surface, and drastically smoothing their spatial distribution. As an example of the limitations of this form of regularization, consider an inversion of the InSAR range changes utilizing the conventional regularization given by equation (4). In this illustration we will use the range changes that occurred between October 2015 and October 2016, shown in the left panel of Fig. 2, as our input data.

We invert for volume changes in a model with six 100 meter thick layers extending from 50 m to 650 m in depth. Each layer is sub-divided into a 45 by 45 grid of rectangular blocks, 2 km on a side, and the model is specified by determining the volume change in each cell of the model. Because we are solving directly for volume changes based upon InSAR observations, and not modeling flow or pressure changes within the aquifer, we adopt simple horizontal layers. That is, the boundaries of our layers are not co-incident with hydrological boundaries and do not reflect the westward deepening basin that dominates the regional hydrogeology^4^ (see Supplementary Fig. S1). However, we do use the textural model^31^, along with mechanical properties associated with the sediments in the San Joaquin Valley^5,32^, to construct a geomechanical model for our interpretation of the InSAR range changes. The model is largely layered and, as all of the layers are unconsolidated sediments, the differences in mechanical properties between the layers is not large. Therefore uncertainties and errors in the model are not likely to have a significant influence on the locations of the volume changes.

**Figure 3:**
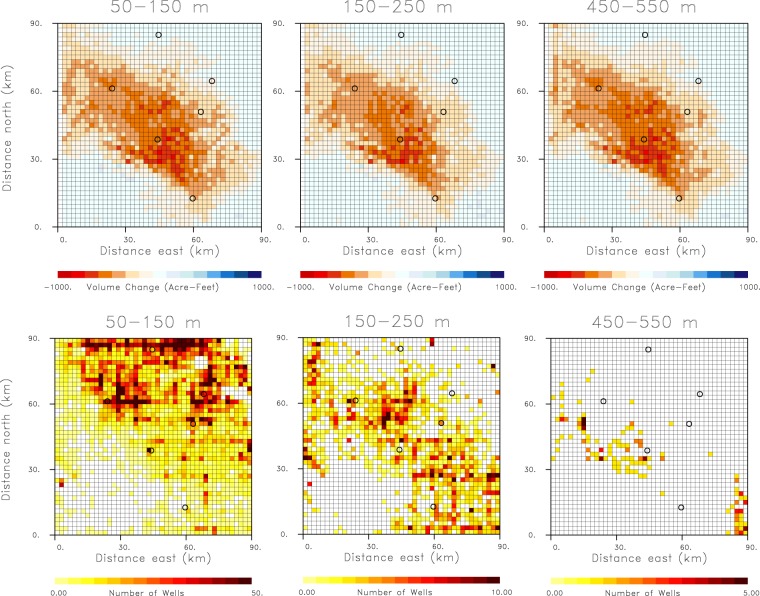
Estimates of aquifer volume change and well distribution. (Upper panels) Estimated spatial variation of the volume changes between October 2015 and October 2016. for three depth intervals in the Tulare basin aquifer model. (Lower panels) Density of wells for three depth ranges within the Tulare basin aquifer model. The open circles in the figure indicate the Central Valley towns that are labeled in the central panel of Fig. 2.

Due to the loss of resolution with depth and the associated non-uniqueness, the inverse problem is unstable and small errors in the data will lead to large errors in the model. In Fig. 3 we plot the estimates of volumes change that minimize the penalized misfit function *P*(v) for three depth intervals within the model. Below the estimated volume changes we display the associated distribution of wells that extend to known depths within the region, obtained from the California Department of Water Resources database. For the most part, the pattern of volume change maintains its form in depth and does not reflect the significant changes in the distributions of wells that we observe between the various layers. This continuity and smoothness is likely due to a combination of the loss of resolution with depth and the regularization that penalizes both rough models and large volume changes at depth.

The conventional set of penalty terms ignores at least one key physical consideration that can be used to constrain the solution set. Specifically, the aquifer volume changes are thought to be driven, in large part, by variations in the effective stress, ***σ***_*e*_,

1 $${{\boldsymbol{\sigma }}}_{e}={{\boldsymbol{\sigma }}}_{t}-{P}_{f}{\bf{I}}$$

due to fluid withdrawal and the associated fluid pressure change *P*_*f*_, from wells distributed throughout the region. The total stress in Eq. (1), ***σ***_*t*_, is typically dominated by the weight of the column of material overlying a particular patch of the aquifer. However, substantial lateral stresses may develop at the edges of subsidence bowls or near active wells. A reduction in fluid pressure due to pumping leads to an increase in effective stress around the well. The increased effective stress induces deformation in the sediments surrounding the well. The amount of deformation can vary greatly depending on the nature of the sediments; sands with well rounded grains generally resist substantial deformation while the platy materials in poorly consolidated clays can rotate and re-arrange, leading to significant deformation^14^. The primary point is that we would expect some correspondence between active wells and volume change in a deforming aquifer.

**Figure 4:**
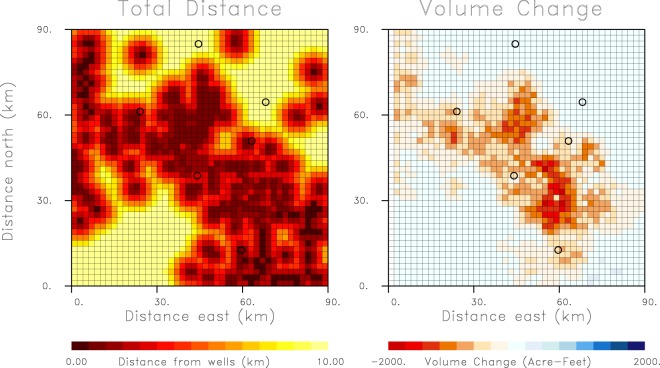
Illustration of well distance penalty for the depth interval 150–250 m in the model. (Left) Well distance measure, with the color representing the distance to the pixels with the nearest set of two or more wells. (Right panel) Results of an inversion for the volume change between October 2015 and October 2016 in the depth interval 150–250 m. The open circles in the figure indicate the Central Valley towns that are labeled in the central panel of Fig. 2.

We can enforce an association between active wells and aquifer volume change in our inversion, through the introduction of a regularization term that penalizes volume changes that are far from any well. This approach has the added benefit that it makes use of one of the few complete and publicly available data sets covering the entire state, the digital map of completed state wells, provided by the California Department of Water Resources. Within the region under study, there are 20,774 wells with known depths that have operated since 2015. The spatial distributions of wells in three layers of the model are shown in Fig. 3, where the number of wells within each grid block are given. The spatial distribution of wells in a given layer may be used to construct a penalty term in order to regularize the inverse problem:

2 $$P({\bf{v}})=\sum _{i}\,{({r}_{i}-{{\bf{g}}}_{i}^{t}{\bf{v}})}^{2}+{W}_{d}{{\bf{v}}}^{t}{\bf{Dv}},$$

where *r*_*i*_ is the i-th range change observations, ${{\bf{g}}}_{i}^{t}$ is a vector containing the impulse response known as the Green’s function components, **v** is the vector of aquifer volume changes, and **D** is a diagonal matrix whose *i*-th entry is equal to the distance of the i-th grid block to the closest grid block that contains two or more wells. In Fig. 4 we plot the penalty terms in the layer of the model between 150 and 250 meters in depth. The penalty terms are zero or small in a diagonal band across the layer corresponding to southeast to northwest trending swath of wells that extend across the Tulare Basin (see Fig. 3). The volume changes minimizing the penalized misfit function (2) are also shown in Fig. 4, corresponding to a distance penalty weighting of *W*_*d*_ = 500. For the most part, the spatial distribution of the volume changes are concentrated near the locations of wells in this depth interval. The pattern of volume change in this layer is very different from the patterns in the earlier inversion that did not contain a well distance penalty (Fig. 3). That model contained a much more wide-spread and smoother spatial distribution, reflecting the roughness and model norm penalties [see equation (4), discussed in the Methods section].

**Figure 5:**
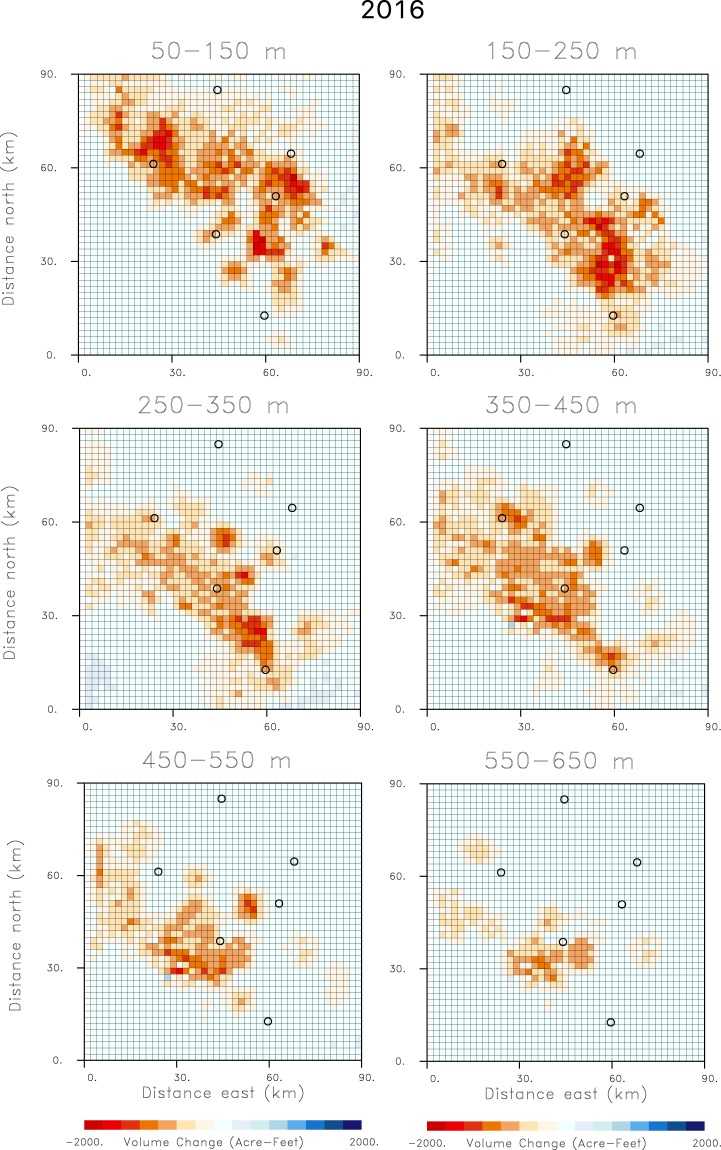
Estimates of volume changes between October 2015 and October 2016, for all of the depth intervals in the aquifer model. The open circles in the figure indicate the Central Valley towns that are labeled in the central panel of Fig. 2.

In order to strike a reasonable balance between fitting the observed range change data and producing a model with volume changes at or near well locations, we constructed a trade-off curve. That is, we conducted a sequence of inversions, increasing the weighting coefficient *W*_*d*_ in the penalty term in expression (2), systematically from 0.01 to 3000.00, and computed the root-mean-squared misfit and the sum of the distance-weighted volume changes. The resulting plot of data misfit versus distance-weighted volume change, for all 151 models used to construct the tradeoff-curve, contains a sharp bend (as shown in Fig. S2 in the Supplement). The points in the bend of the curve represent models that do not have excessive misfit nor large volume changes far from existing wells. It was found that a representative model in the bend of the curve had a weight *W*_*d*_ of 500. This weight resulted in good correspondence between the volume changes and the well locations, and a good fit to the observed range change. The estimated volume changes for the six layers of the model are shown in Fig. 5 for the water year from October 2015 until October 2016. The spatial distribution of volume decrease changes significantly with depth, reflecting the variations in the wells that reach each layer. With depth there is a progressive shift from wells located in the north and east to wells located to the southwest. This reflects the deepening of the basin to the southwest that is evident in the topography of the base of the aquifer (see the Supplementary Fig. S1). In some intervals, such as the depth range 250 to 350 m, areas of volume change appear to follow linear trends, suggesting some form of structural control.

**Figure 6:**
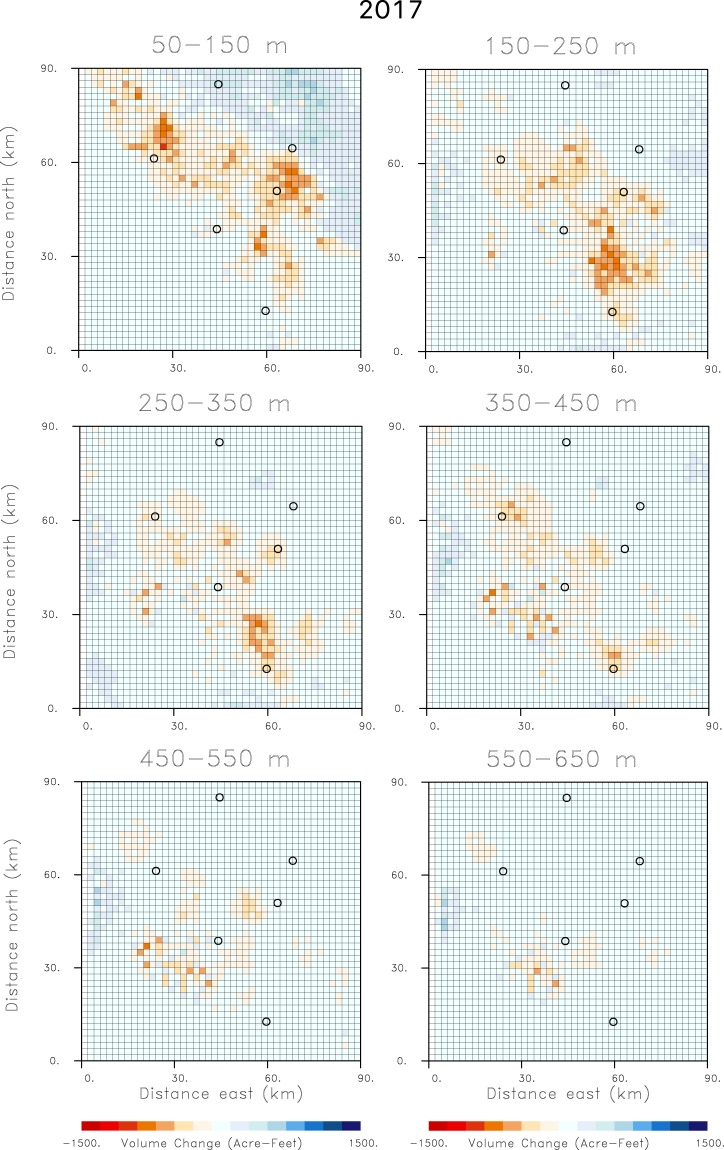
Estimates of volume changes between October 2016 and October 2017, for all of the depth intervals in the aquifer model. The open circles in the figure indicate the Central Valley towns that are labeled in the central panel of Fig. 2.

**Figure 7:**
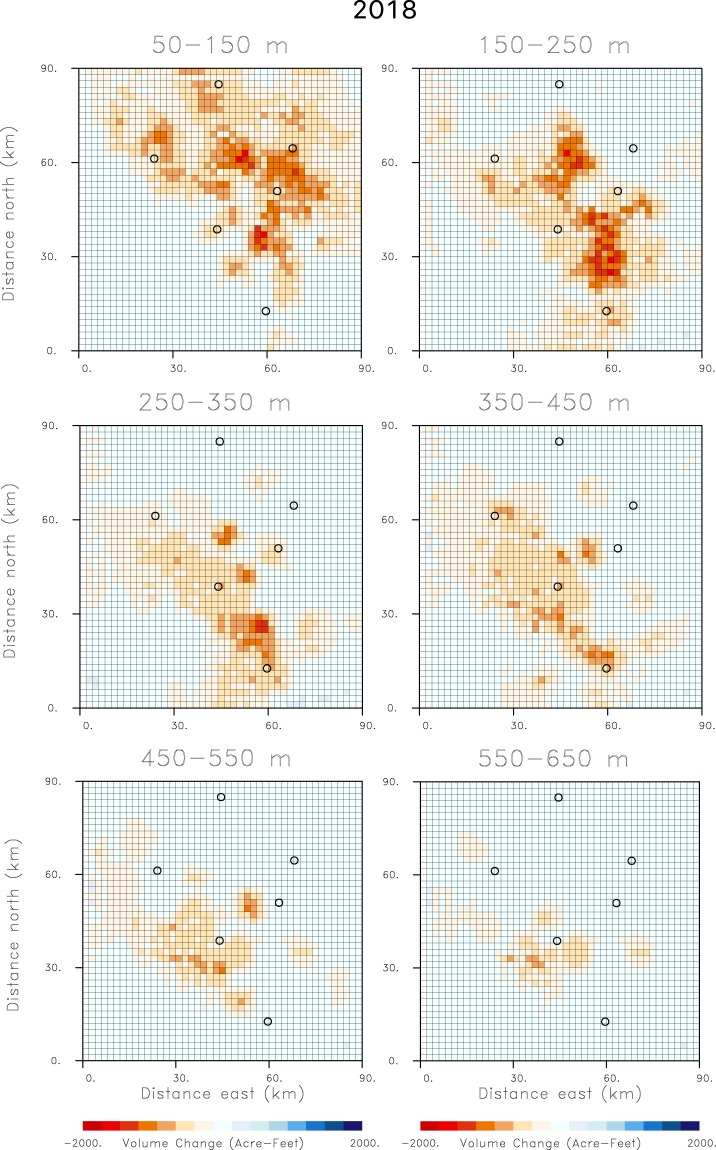
Estimates of volume changes between October 2017 and October 2018, for all of the depth intervals in the aquifer model. The open circles in the figure indicate the Central Valley towns that are labeled in the central panel of Fig. 2.

The time interval from October 2015 to October 2016 was a period of drought in California, characterized by extensive withdrawal of groundwater. Hence, there is significant subsidence, indicated in Fig. 2, and large volume decreases of over 2000 acre-feet for the grid blocks shown in Fig. 5. Over the next water year, from October 2016 until October 2017, California experienced significant rainfall leading to increased water deliveries and water availability. The additional supplies of surface water led to decreased groundwater pumping and a reduction in aquifer compaction and less subsidence. In fact, there are shallow volume increases in the northeast corner of the top-most layer, perhaps signifying elastic rebound due to recharge from the foothills of the Sierra Nevada range (Fig. 6). The decreased subsidence is documented in Fig. 2 as significantly less range change than in 2016. Similarly, the estimated volume changes between October 2016 and October 2017, shown in Fig. 6, are much smaller than those of the previous year. There appears to be very little deep volume change, suggesting a substantial reduction in groundwater pumping from deep wells. During the following year, from October 2017 to October 2018, California experienced moderate rainfall, more in-line with historical averages. Correspondingly, the range change indicates subsidence values between those of 2016 and 2017. The pattern of volume change for 2018, plotted in Fig. 7, is somewhat similar to that of 2016 but of much smaller magnitude. Notably, the volume decreases at the northernmost end of the area are larger than those in 2016 (Fig. 5). This might be an indication of increased pumping from replenished shallow aquifers, as supported by the rebound in 2017 (Fig. 6).

**Figure 8:**
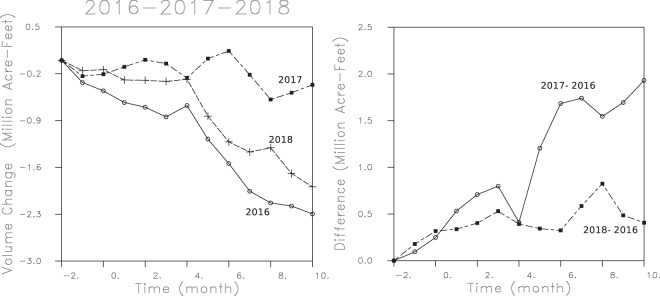
(Left panel) Total aquifer volume change for three one year intervals from October of the preceding year to October of that year. The total volume change is obtained by summing the volume changes of every grid block in the model. (Right panel) Difference between cumulative changes for the water years 2017 and 2018, and those of the water year 2016. That is, the volume changes during 2016 are subtracted from those of 2017 and 2018 in order to derive these two curves.

One can make use of the favorable temporal sampling of the Sentinel 1a/b satellites, as they produce a SAR image of the Tulare basin every 6 to 12 days, to examine the volume changes at monthly intervals. To this end, the range changes were interpolated into monthly changes and these were inverted for three-dimensional models of volume change, similar to those shown in Figs 5–7. The volume changes in every grid block were summed to get a total volume change for the Tulare basin during that month. In the left panel of Fig. 8 we plot the cumulative volume change for three different one year intervals from October of the preceding year to October of the listed year. The monthly changes accumulate for each year to give the total aquifer volume change for each of the three years. There is a dramatic decline for the year 2016, capturing the effect of the continuing drought over that time interval. Following that water year, during the wet period from October 2016 to October 2017, the loss of aquifer volume declined dramatically. The accumulating reduction in aquifer volume is highlighted in the right panel of Fig. 8, where we plot the difference in volume changes from that year, referenced to the previous year of 2016. The differences between the years are quite substantial and suggest a rapid response of the aquifer system to probable changes in pumping rates. Note however, there is a general decrease in volume even during the wet year from October 2016 to October 2017. This trend may reflect continuing inelastic volume change initiated by pumping from earlier years. The changes are less dramatic when we compare 2016 and 2018, but there is still over 0.4 Million acre-feet less of aquifer volume that was lost during 2018.

## Discussion

In an effort to link aquifer volume change to the distribution of existing wells in the Tulare Basin we have introduced an inversion technique that penalizes models containing significant volume change in areas far from known well locations. Using this approach we are able to produce spatial distributions of volume change that are co-located with documented wells, in contrast to a conventional inversion without the well-distance penalty. Adding the well-distance penalty does not seem to degrade the fit to the observations. In fact, inversions with and without the well distance penalty both give variance reductions of 99.6%, where the variance is defined as the sum of the squares of the difference between the *M* observations and range change *r*_*i*_ and their calculated values *c*_*i*_,

$${\sigma }^{2}=\frac{1}{M}\mathop{\sum }\limits_{i\mathrm{=1}}^{M}\,{({r}_{i}-{c}_{i})}^{2}.$$

The variation reduction is the ratio of the difference between the initial (*σ*_*i*_) and final (*σ*_*f*_) variance to the initial variance: (*σ*_*i*_ − *σ*_*f*_)/*σ*_*i*_ given as a percentage. Both approaches produce post-inversion residual distributions that are much smaller than the observed line-of-sight displacements shown in Fig. 2. The final standard deviations of the residual distributions are 0.81 cm and 0.94 cm, for the inversions without and with the well-distance penalty, respectively. These values are both close to the estimated error of 1 cm associated with the line-of-sight observations. So it appears that the presence of the well-distance penalty and the requirement that the volume change occur in the vicinity of wells is compatible with the observed InSAR data.

Though the aquifer volume change is only a lower bound on the amount of water lost in the Tulare Basin it is useful to compare the values plotted in Fig. 8 to the loss of aquifer storage estimated from other studies. The total loss of aquifer volume of 2.3 million acre-feet between October 2015 and October 2016 agrees with calculated values of the cumulative annual change in aquifer storage for the Tulare basin produced by the Central Valley Hydrologic Model^4^ (Figure B9). The value agrees with an estimated yearly loss of 2.3 million acre-feet of groundwater, presented by the Friant Water Authority. It is of the same order as the 1.8 million acre-feet/year of groundwater overdraft estimated by the Public Policy Institute of California^33^. Because the groundwater loss in the Central Valley is dominated by losses in the Tulare basin^4^, our estimate of volume decrease, 2.9 cubic kilometers/year, is also compatible with the 3.1 cubic kilometers/year (2.5 million acre-feet/year) loss in groundwater volume for the entire Central Valley of California, determined from gravitational variations detected by the Gravity Recovery and Climate Experiment (GRACE) satellite mission^1^ for the years 2003–2010. The gravity observations are sensitive to the total water mass change, including variations in groundwater storage as well as changes in the water mass due to water table variations. The GRACE data does not extend to our more recent time intervals, but the rate of groundwater loss appears to be roughly linear from about 2006 until 2015^9^. The estimated groundwater volume change between October 2016 to October 2017 is significantly smaller, 0.3 million acre-feet/year, a reflection of the significant rainfall and increased water deliveries that year, which appear to have sharply curtailed groundwater pumping^17^. The groundwater volume change for the succeeding year, a loss in volume of 1.6 million acre-feet/year, is roughly midway between these two extremes. The generally yearly pattern for 2016 and 2018 contains a sharp decrease in groundwater volume in May, June, and July, signifying greater pumping, perhaps due to decreased water deliveries during those months. The pattern is disrupted for the wet year of 2017, with a decrease in groundwater loss between April and May.

**Figure 9:**
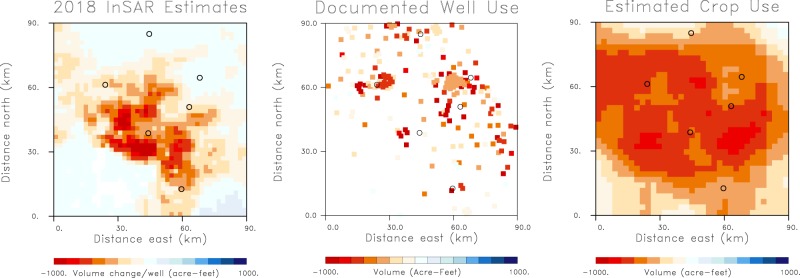
(Left panel) Volume change per well obtained from InSAR range change observations corresponding to the water year 2018, a year with rainfall near the historical average. (Center panel) Water usage for a selected set of wells with measured flow meters. (Right panel) Estimated yearly water usage over the Tulare basin area calculated from the distribution of crops and the water needs of each plant or animal.

This work is the first application of such a constrained inversion for aquifer volume change and there are limitations to this work, primarily the fact that we can only estimate a lower bound on water usage. However, there is also the potential for significant enhancements to this approach. First, we can incorporate other information as it becomes available and reliable, such as well pumping rates and volumes, and estimates of water use based on known crop distributions^5^, as is used in the Central Valley hydrological model^4^. In the left panel of Fig. 9 we plot estimates of volume change per well for the water year 2018, obtained from our results by dividing the volume change in each grid block by the number of wells intersecting the grid block. In the central panel of Fig. 9 we present the yearly water volumes extracted by each well, for the few wells where there are measured flow rates. Because the rainfall during 2018 was close to historical averages, we can compare these values to the estimated yearly water usage over the region obtained from the known crop distributions and estimates of the water needs of each crop^5^, as shown in the right panel of Fig. 9. There is general agreement between the yearly water usage estimated from InSAR and observed in wells, with peak values near 2000 acre-feet/year. There is also a correspondence between high usage areas, estimated from crop distributions, and regions displaying significant volume change per well. We can incorporate other data sets such as gravity data as a constraint on the inversion, though that will require accurate modeling in order to apply all of the necessary corrections^1^. In addition, we can add other observations such as extensiometers and water level data^32^. Better modeling, including improved estimates of aquifer properties will allow for direct estimates of water volumes and inelastic storage, taking the next difficult step. Applying the approach to a long historical record will allow for the determination of long and short term trends in the volume changes and to estimates of the elastic and inelastic contributions.

An advantage of our approach, one that is particularly important for water managers and responsible officials, is the ability to investigate changes in water resources at various scales. This study was conducted at a large scale in order to encompass the entire Tulare basin. This scale is appropriate for large scale basin assessments and for California state reporting by hydrologic region. However, California’s recently implemented groundwater management law states that long term viability of water resources should be assessed at the scale of a Groundwater Sustainability Agency (GSA). These GSAs vary in size greatly but are generally smaller than a full hydrological basin. However, the general approach described in this study can be conducted at a much smaller scale^20^. For example, one can examine areas of a few square kilometers in size, with grid block dimensions of tens to hundreds of meters as was done for oil and gas applications. This means that a similar approach could be used to assess the resources of a given GSA, and, very importantly, exactly the same approach can be used to evaluate the resources of a neighboring GSA. This would allow consistent and uniform metrics of resource assessment between agencies and throughout a connected basin. Furthermore, studies with higher resolutions of tens to hundreds of meters will be useful in monitoring groundwater recharge and water-banking efforts, activities that are likely to become increasingly important, due to climate change. These more detailed investigations can make use of water-level, infiltration, and pumping rate data, in addition to well locations, in order to quantify fluid volume changes and the migration of injected or ponded water. Such monitoring, in conjunction with a detailed groundwater modeling code, will allow for the calculation of water balances and groundwater sustainability. The fine time resolution of the InSAR observations enables water managers to assess the impact of particular recharge events, management actions or storm events, in order to determine efficacy and to improve best practices. In conclusion, the availability of a consistent approach for resource assessment from project to basin scale that can be updated with fine temporal resolution creates the potential for a widely adoptable standard of measurement that would greatly benefit the rational and sustainable management of groundwater resources.

## Methods

Synthetic Aperture Radar (SAR) imaging, provides one of the most cost-effect techniques for estimating surface deformation over wide areas (hundreds of square kilometers), at a fine spatial resolution (tens of meters), and, under favorable conditions, with centimeter-level accuracy^34^. In this approach a microwave chirp, sent from an orbiting satellite, is reflected back from scatterers on the Earth’s surface and the complex return trace is recorded and processed. In the interferometric mode SAR observations from a series of satellite passes are used to calculate phase changes for a sequence of time intervals. The phase shifts are influenced by several factors, including atmospheric phase delays, orbital errors, incomplete topographic corrections, changes in the reflective properties of the Earth’s surface, and range change, the quantity of most interest for monitoring groundwater-related subsidence^34–36^. Range is the distance to the satellite, and range change provides a component of displacement that is sensitive to the subsidence or uplift of the Earth’s surface as well as to the horizontal components of motion. Using many interferograms in conjunction with spatial and temporal filtering, along with the use of a stable reference location, one can mitigate the effects of atmospheric variations that contaminate the phase^34^. The utility of InSAR monitoring is aided by the freely available SAR observations gathered over California by the European Space Agency’s Sentinel-1a/b satellite pair, providing repeat coverage every 6 to 12 days. The dense temporal sampling reduces the de-correlation that can occur between successive SAR acquisitions, an important factor in the largely agricultural Central Valley, where changes in the surface characteristics of a field can alter the reflective properties of the surface^15,17^. The temporal sampling is also advantageous in the application of the Small Baseline Subset^37,38^ approach for constructing time series of surface deformation on a pixel by pixel basis. In this approach interferometric pairs with small spatial (perpendicular) baselines and short temporal separations are used to estimate the line-of-sight displacements that constitute the range changes. Data from 98 SAR images, from January 31, 2015 until November 17, 2018, were processed using the Interferometric Scientific Computing Environment (ISCE) from the Jet Propulsion Laboratory (JPL), for implementing the Small Baseline Subset approach^39^ and deriving a field of range change times series for much of the Tulare Basin.

We outline the method used to estimate the aquifer volume changes that are compatible with a given set of observed range changes. It is assumed that outside of those regions undergoing volume change, the Earth behaves in an elastic fashion over the time intervals between SAR acquisitions. We parameterize the aquifer model by sub-dividing it into a set of non-overlapping grid blocks. Each grid block may undergo a change in volume, driven by the withdrawal of water from a well or an increase in aquifer pressure due to fluid influx. The deforming aquifer, the source grid blocks, may behave in an arbitrarily complicated fashion, including viscoelastic and plastic behavior. In particular, the material within a grid block may experience elastic and inelastic deformation due to compaction produced by groundwater withdrawal^3,29^. However, because the overburden and adjacent regions are assumed to respond to the volume change within the source region in an elastic manner, the observed deformation at a point ***x***_*i*_ on the Earth’s surface, denoted by *u*_*r*_(**x**_*i*_), is linearly related to the volume change associated with the *n*-th grid block in the aquifer model, *v*(**r**_*n*_),

3 $${u}_{r}({{\bf{x}}}_{i})=\mathop{\sum }\limits_{n\mathrm{=1}}^{N}\,{\Gamma }_{in}v({{\bf{r}}}_{n})={{\bf{g}}}_{i}^{t}{\bf{v}}$$

where Γ_*in*_ is the elastic Green’s function, the response function for a rectangular grid block, centered at **r**_*n*_, and the summation is over all *N* of the grid blocks in the aquifer model^18–20^. The components of the indexed vector **g**_*i*_ are the response functions for each of the rectangular blocks in the model, while the vector **v** contains their fractional volume changes. The complexity of the Green’s function calculations depend upon the elastic properties of the region. For a highly symmetric elastic model, such as a homogeneous half-space, there are analytic expressions^40–42^ for simple sources such as a point or a rectangular grid block. More elaborate elastic structures require semi-analytic or fully numerical methods. For example, the Green’s function for a layered elastic or viscoelastic medium can be computed semi-analytically^43^ while a fully three-dimensional medium requires numerical methods, such as a finite-difference or finite-element approach^44–46^. In our implementation we used an averaged one-dimensional elastic structure provided by data from exploration wells.

The inverse problem is formulated as a least-squares optimization problem in which we seek a spatial distribution of volume changes minimizing the sum of the squares of the InSAR range change residuals^18–20^. In order to stabilize the inversion in the face of the non-uniqueness, one typically introduces penalty or regularization terms that minimize some aspect of the model, such as the size of the volume changes or the overall roughness of the model^30^. The penalty terms are most often formulated as quadratic measures such as the sum of the squares of the volume changes, the model norm, or the sum of the squares of the spatial gradient of the model, the model roughness, leading to the minimization of a function such as

4 $$P({\bf{v}})=\sum _{i}\,{({r}_{i}-{{\bf{g}}}_{i}^{t}{\bf{v}})}^{2}+{W}_{n}{{\bf{v}}}^{t}{\bf{v}}+{W}_{r}{{\bf{v}}}^{t}{{\bf{L}}}^{t}{\bf{L}}{\bf{v}}$$

where *r*_*i*_ is the *i*-th range change observation, **g**_*i*_ is the Green’s function vector associated with the *i*-th observation, *W*_*n*_ is a coefficient that determines the weight given to the model norm penalty, *W*_*r*_ is the model norm roughness weight, and **L** is a matrix that approximates the spatial gradient operator^30^. In the work described above we use the alternative formulation (2) where the distance to the wells **D** is used for the regularization. The condition for a minimum of *P*(**v**), obtained by setting the gradient with respect to the model parameters (${\nabla }_{{\rm{v}}}P$) to zero, produces a linear system of equations that may be solved for **v**, in our case using the least squares QR algorithm^47^. The weighting coefficients can be chosen by trial and error or through the construction of a multidimensional trade-off curve. For the misfit function introduced in this study, that includes the well distance penalty given by Eq. (2), we constructed a trade-off curve, as discussed above and shown in Fig. S2.

## Supplementary information

Supplementary Figures for the paper
