# Supplementary material for: Satellite-based monitoring of groundwater depletion in California’s Central Valley
Source: Sci Rep. 2019 Nov 5;9:16053. doi: 10.1038/s41598-019-52371-7 (PMC6831828; doi:10.1038/s41598-019-52371-7)
Supplement: Supplementary file 1 — Supplementary Figures for the paper [file 41598_2019_52371_MOESM1_ESM.pdf]

Satellite-based monitoring of groundwater depletion in California's Central Valley

D. W. Vasco, Tom G. Farr, Pierre Jeanne, Christine Doughty, and Peter Nico

October 7, 2019

Supplementary Materials

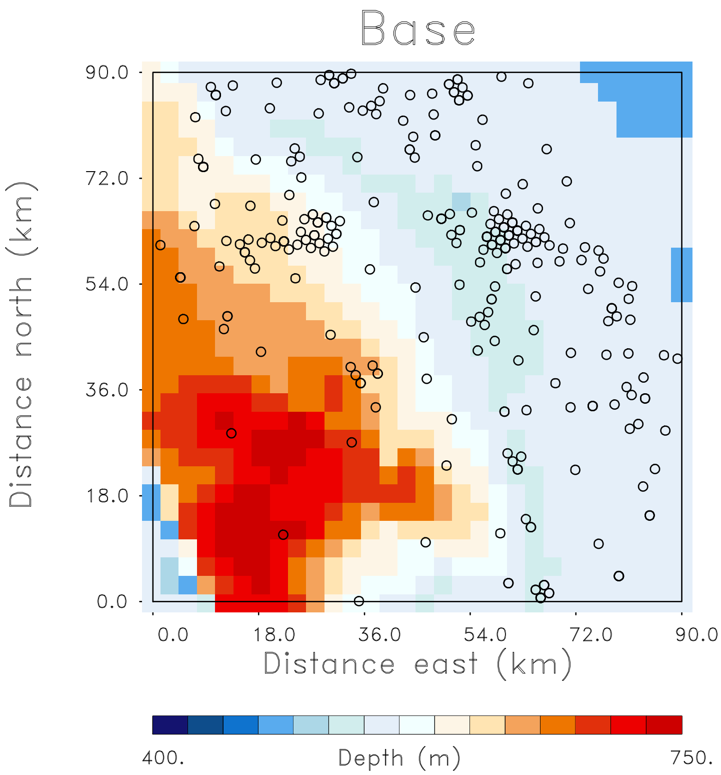

Figure S 1: Topography of the base of the Central Valley aquifer model in the vicinity of the Tulare Basin. The open circles denote a representative sampling of wells in the region.

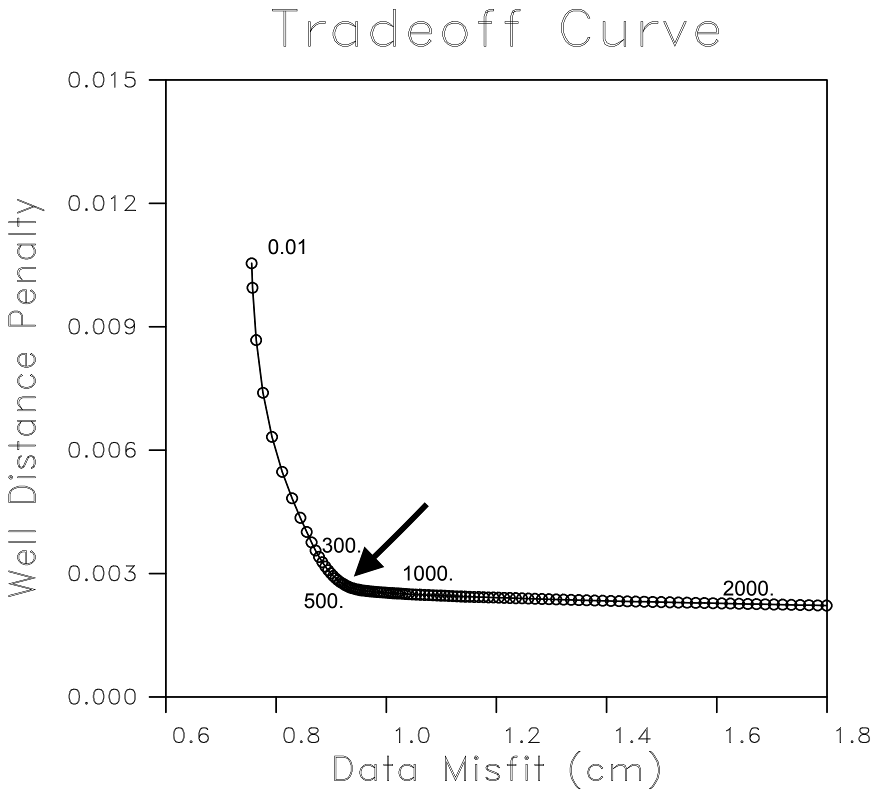

Figure S 2: Tradeoff-curve obtained by conducting 151 inversions with varying values of the weighting coefficient W_d that appears in the penalized misfit function given by equation (2). The arrow indicates the location of the weighting used in the inversion of the actual observations.

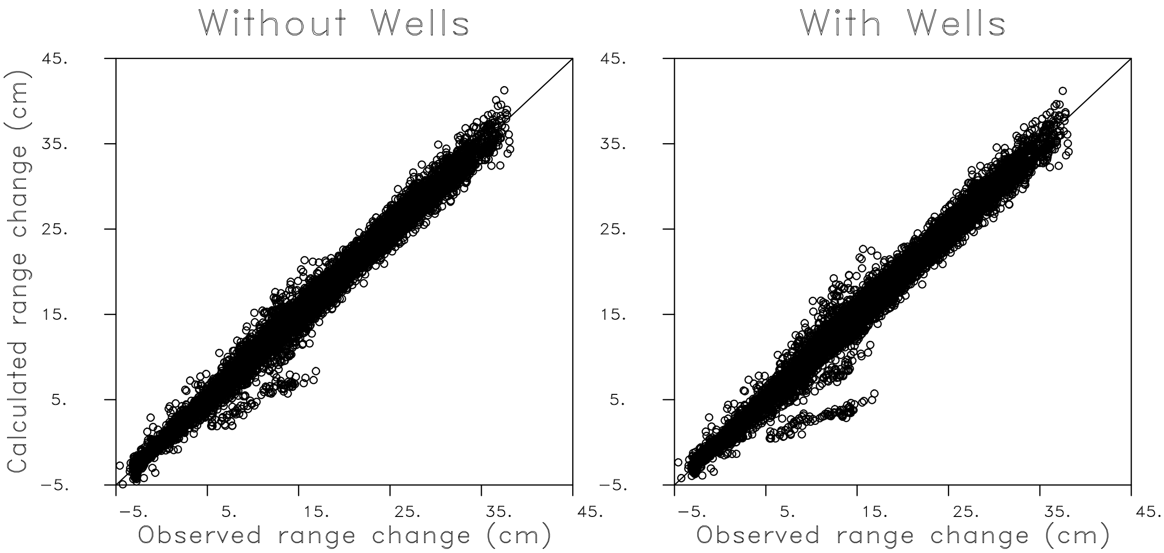

Figure S 3: Observed range change plotted against range change predicted using the solutions to the inverse problems for volume changes. (Left panel) Least squares solution minimizing equation (4), with no penalty for volume change away from known wells. (Right panel) Least squares solution minimizing equation (2), with a penalty for volume change far from existing wells.
